# Non-T-depleted haploidentical transplantation with post-transplant cyclophosphamide in patients with secondary versus de novo AML in first complete remission: a study from the ALWP/EBMT

**DOI:** 10.1186/s13045-023-01450-4

**Published:** 2023-05-29

**Authors:** Arnon Nagler, Myriam Labopin, Didier Blaise, Anna Maria Raiola, Lucia Lopez Corral, Stefania Bramanti, Simona Sica, Mi Kwon, Yener Koc, Jiri Pavlu, Alexander Kulagin, Alessandro Busca, Arancha Bermúdez Rodríguez, Péter Reményi, Christoph Schmid, Eolia Brissot, Jaime Sanz, Ali Bazarbachi, Sebastian Giebel, Fabio Ciceri, Mohamad Mohty

**Affiliations:** 1 Division of Hematology, Sheba Medical Center, Tel Hashomer, Israel; 2 EBMT Paris Study Office, Department of Haematology, Saint Antoine Hospital; INSERM UMR 938, Sorbonne University, Paris, France; 3 Department of Haematology, Saint Antoine Hospital, INSERM UMR 938, Sorbonne University, Paris, France; 4 Programme de Transplantation and Therapie Cellulaire Centre de Recherche en Cancérologie de Marseille, Institut Paoli Calmettes, Marseille, France; 5 Ematologia e Terapie Cellulari, IRCCS Ospedale Policlinico San Martino, Genova, Italy; 6 Hospital Clínico Servicio de Hematología, Salamanca, Spain; 7 Transplantation Unit Department of Oncology and Haematology, Istituto Clinico Humanitas, Milan, Italy; 8 Dipartimento di Diagnostica per Immagini, Radioterapia Oncologica ed Ematologia, Fondazione Policlinico Universitario A. Gemelli IRCCS, Rome, Italy; 9 Sezione di Ematologia, Dipartimento di Scienze Radiologiche ed Ematologiche, Università Cattolica del Sacro Cuore, Rome, Italy; 10 Hematology Hospital GU Gregorio Marañon, Instituto de Investigacion Sanitaria Gregorio Marañon, Medicina UCM, Madrid, Spain; 11 Bone Marrow Transplant Unit, Medicana International Hospital Istanbul, Istanbuls, Turkey; 12 Department of Haematology, Hammersmith Hospital, Imperial College, London, UK; 13 Raisa Gorbacheva Memorial, Research Institute for Paediatric Oncology, Hematology, and Transplantation, First State Pavlov Medical University of St. Petersburg, St. Petersburg, Russia; 14 SSD Trapianto di Cellule Staminali, AOU Citta’ Della Salute e della Scienza, Turin, Italy; 15 Hospital U. Marqués de Valdecilla, Servicio de Hematología-Hemoterapia, Santander, Spain; 16 Dél-pesti Centrumkórház – Országos Hematológiai és Infektológiai Intézet, Department Hematology and Stem Cell Transplant, Budapest, Hungary; 17 Department of Hematology and Oncology, Augsburg University Hospital, Augsburg, Germany; 18 Service d’Hématologie Clinique et Thérapie Cellulaire, Hôpital Saint-Antoine, AP-HP, Sorbonne University, and INSERM UMRs 938, Paris, France; 19 Hematology Department, Hospital Universitari Politècnic La Fe, Valencia, Spain; 20 Bone Marrow Transplantation Program, Department of Internal Medicine, American University of Beirut, Beirut, Lebanon; 21 Department of Bone Marrow Transplantation and Onco-Hematology, Maria Sklodowska-Curie National Research Institute of Oncology, Gliwice Branch, Gliwice, Poland; 22 Ospedale San Raffaele, Haematology and BMT, Milan, Italy

**Keywords:** Haploidentical allogeneic stem cell transplantation, Post-transplantation cyclophosphamide, Secondary acute myeloid leukemia, De novo acute myeloid leukemia, Transplantation outcomes

## Abstract

We compared outcomes of adult patients with secondary acute myeloid leukemia (sAML) versus de novo AML after non-T-depleted haploidentical stem cell transplant (HaploSCT) with post-transplant cyclophosphamide (PTCy). Seventeen hundred and eleven AML patients (sAML-231, de novo-1480) in first complete remission transplanted from 2010 to 2021, were included. Patients with de novo AML were younger, median age 55.8 versus 60.8 years, *p* < 0.0001, had better transplantation comorbidity index (HCT-CI) ≥ 3 21.3% versus 40.8%, *p* < 0.0001 and Karnofsky performance status (KPS) with KPS ≥ 90 in 78% versus 68.5%, respectively, *p* = 0.002. The two patient groups did not differ with respect to gender, cytomegalovirus serostatus, and cell source. Median time from diagnosis to HaploSCT was 5.2 versus 4.9 months, respectively, *p* = 0.005. Fewer sAML patients received myeloablative conditioning 35.1% versus 50.1%, *p* < 0.0001. Two hundred and eleven sAML and 410 de novo AML patients were included in the matched-pair analysis matching two de novo AML with each sAML. No significant difference was observed in any transplantation outcome parameter between the sAML versus de novo AML groups. Two-year non-relapse mortality and relapse incidence did not differ with HaploSCT for de novo versus sAML; 21.4% versus 21%, hazard ratio (HR) = 0.98, *p* = 0.9 and 23.4% versus 20.6%, HR = 0.92, *p* = 0.67, respectively. Two-year leukemia-free survival, overall survival, and graft-versus-host disease (GVHD)-free, relapse-free survival were also not different between the de novo AML and sAML groups 55.2% versus 58.4%, HR = 0.95, *p* = 0.67; 61.4% versus 66.4%, HR = 0.91, *p* = 0.51 and 46.3% versus 48.2%, HR = 0.92, *p* = 0.48, respectively. Similarly, the incidence of engraftment as well as acute and chronic GVHD was similar between the 2 cohorts. In conclusion, HaploSCT with PTCy may be able to overcome the bad prognosis of sAML as results are not significantly different to those of HaploSCT in de novo AML.

**Supplementary Information:**

The online version contains supplementary material available at 10.1186/s13045-023-01450-4.

## Introduction

Secondary acute myeloid leukemia (sAML) is a distinct type of acute myeloid leukemia (AML) evolving from an antecedent hematological disorder or as a complication of prior cytotoxic chemotherapy or radiation therapy [1, 2]. Patients with sAML have inferior outcomes compared to de novo AML, mainly due to a higher frequency of adverse molecular mutations and high-risk cytogenetic abnormalities in addition to typically being older and having an antecedent hematological disease [3–7]. Allogeneic hematopoietic cell transplantation (HSCT) represents a potentially curative therapy in this setting, rescuing up to 40% of the patients [8–12]. Despite some improvement in matched sibling and unrelated transplantation for sAML in the last few decades, as we have recently demonstrated on behalf of the Acute Leukemia Working Party (ALWP) of the European Society for Blood and Marrow Transplantation (EBMT) with a study of sAML patients comparing 1337 that were transplanted in 2000 to 2010 with 2887 transplanted in 2011 to 2020. We demonstrated a significant reduction in 2-year non-relapse mortality (NRM) and a significant improvement in the 2-year graft-versus-host disease (GVHD)-free, relapse-free survival (GRFS) but the 2-year leukemia-free (LFS) and overall survival (OS) were similar [13] with somewhat better results with myeloablative (MAC) versus reduced intensity conditioning (RIC) [9, 14]. These results are better than those reported in 2010 by the Center for International Blood and Marrow Transplant Research (CIBMTR) in 868 patients with therapy-related AML or myelodysplastic syndrome (MDS) transplanted between 1990 and 2004 mainly from matched sibling donors (MSD) or matched unrelated donors (MUD) and MAC with 5-year disease-free survival (DFS) and OS of 21% and 22%, respectively, with the caveat that the CIBMTR study included also patients in second CR and more advance disease [8], or our previous results evaluating transplantation outcome in close to 5000 patients with sAML transplanted between 2000 and 2016 mainly from MSD and MUD, where we observed 2-year OS, LFS and GRFS of 44.5%, 38.8% and 27.2%, respectively [9]. Notably, transplantation outcomes with MSD and MUD in sAML are significantly inferior to those typically achieved in de novo AML with a lower OS, LFS, and GRFS due to higher NRM and relapse incidence (RI) [10]. However, the picture may differ with non-T depleted haploidentical stem cell transplantation (HaploSCT) with post-transplant cyclophosphamide (PTCy) which has been increasingly used for AML and proven to be highly effective in preventing GVHD and reducing NRM thus improving transplantation results [15, 16]. HaploSCT for sAML has been performed in recent years [15, 16] with a 2-year LFS of 49% and OS of 57% in patients transplanted in complete response (CR) from 2006 to 2016 [15]. Furthermore, some reports indicate a stronger graft-versus-leukemia (GVL) effect with Haplo grafts due to the broad human leukocyte antigen (HLA) disparity [19, 20] which may be of special importance in sAML being a high-risk leukemia category carrying a high post-transplantation RI [8]. Indeed, relapse is the most frequent cause of transplant failure in sAML with a poor prognosis, a median OS of about 8 months, and limited therapeutic options [8–10, 21, 22]. It is conceivable therefore that the results of HaploSCT in sAML will not differ from those in de novo AML. Such a comparison has not yet been performed. Therefore, the goal of the current study was to compare the outcomes of HaploSCT in patients with sAML with those of HaploSCT in de novo AML.

## Patients and methods

### Study design and data collection

This was a retrospective, multicenter analysis using the dataset of the ALWP of the EBMT. The EBMT is a voluntary working group of more than 600 transplant centers that are required to report all consecutive stem cell transplantations and follow-ups once a year. EBMT minimum essential data forms are submitted to the registry by transplant center personnel following written informed consent from patients in accordance with the centers’ ethical research guidelines. Data accuracy is assured by the individual transplant centers and by quality control measures such as regular internal and external audits. In addition, the study protocol was approved by each site and complied with country-specific regulatory requirements. The results of disease assessments at HSCT were also submitted and form the basis of this report. Eligibility criteria for this analysis included adult patients ≥ 18 years of age with de novo or sAML in CR1 who underwent the first HSCT from a non-T-cell depleted Haplo donor with PTCy as part of GVHD prophylaxis between 2010 and 2021. A Haplo donor was defined as ≥ 2 HLA mismatches between donor and recipient. The exclusion criteria were HSCT from other donor types (sibling, unrelated, or cord blood donor); previous history of HSCT, T cell-depleted hematopoietic cell graft, unknown or favorable cytogenetic risk and unknown antecedent hematological disorder. Data collected included recipient and donor characteristics (age, gender, cytomegalovirus (CMV) serostatus, Karnofsky performance status (KPS) and hematopoietic cell transplantation specific comorbidity index (HCT-CI), disease characteristics, antecedent hematological disorder, year of transplant, type of conditioning regimen, stem cell source, and GVHD prophylaxis regimen. The conditioning regimen was defined as MAC when containing total body irradiation (TBI) with a dose > 6 Gray or a total dose of busulfan (Bu) > 8 mg/kg or > 6.4 mg/kg when administered orally or intravenously, respectively. All other regimens were defined as RIC [23]. Grading of aGVHD was performed using established criteria [24]. Chronic (c) GVHD was classified as limited or extensive according to published criteria [25]. For this study, all necessary data were collected according to the EBMT guidelines, using the EBMT minimum essential data forms. The list of institutions contributing data to this study is provided in the Additional file 1: Appendix.

### Statistical analysis

The median, interquartile range (IQR), and range were used for quantitative variables, and frequency and percentage for categorical variables. The study endpoints were OS, LFS, RI, NRM, engraftment, aGVHD, cGVHD, and GRFS. All endpoints were measured from the time of transplantation. Engraftment was defined as achieving an absolute neutrophil count of 0.5 × 10^9^/L for three consecutive days. OS was defined as time to death from any cause. LFS was defined as survival with no evidence of relapse or progression. NRM was defined as death from any cause without previous relapse or progression. We used modified GRFS criteria. GRFS events were defined as the first event among grade III-IV aGVHD, extensive cGVHD, relapse, or death from any other cause [26]. Patient, disease, and transplant-related characteristics for the two cohorts (de novo and secondary AML) were compared using the Mann–Whitney *U* test for numerical variables, and the chi-squared or Fisher’s exact test for categorical variables. The probabilities of OS, LFS, and GRFS were calculated using the Kaplan–Meier estimate. The RI and NRM were calculated using cumulative incidence (CI) functions in a competing risk setting, with death in remission being treated as a competing event for relapse. Early death was considered as a competing event for engraftment. To estimate the CI of acute or cGVHD, relapse and death were considered as competing events. Univariate analyses were performed using the log-rank test for LFS and OS while Gray’s test was used for CI. Multivariate analyses were performed using the Cox proportional-hazards regression model [27]. All variables differing significantly between the two groups, and potential risk factors were included in the model. In order to take into account, the heterogeneity in the effect of a characteristic or a treatment across centers, we introduce a random effect (also named frailty effect) in Cox multivariate models. Then, the same random effect is shared by all patients within the same center [28].

For each patient with secondary AML, two separate matched controls with de novo AML were identified using exact and propensity-score matched criteria. Exact matching was used for cytogenetics risk group, conditioning intensity, source of stem cells and sex matching (female to male vs all others), and nearest neighbor for recipient age, and Karnofsky score (90–100 vs < 90) [29]. HCT-CI was not included in the propensity score because of the high number of missing values.

Comparisons were performed using a Cox model and cluster-robust standard errors were used to account for dependence between observations within matched pairs. Results were expressed as the hazard ratio (HR) with a 95% confidence interval (95% CI). All *p* values were two-sided with a type 1 error rate fixed at 0.05. Statistical analyses were performed with SPSS 25.0 (SPSS Inc., Chicago, IL, USA) and R 4.0.2 (R Core Team Fifty (2020). R: A language and environment for statistical computing. R Foundation for Statistical Computing, Vienna, Austria. URL https://www.R-project.org/) [30].

## Results

### Patient, transplant, and disease characteristics

A total of 1711 patients met the inclusion criteria, 231 with sAML and 1480 with de novo AML. Table 1 shows the baseline demographic and clinical characteristics. Median follow-up was 24.6 (IQR 19.6–31.2) and 26.3 (IQR 24.5–28.8) months for patients with sAML and de novo AML, respectively (*p* = 0.52). Patients with de novo AML were younger, with a median age of 55.8 (range 18.1–82.5) versus 60.8 (20.8–75.7) years, (*p* < 0.0001). The median year of transplant was 2019 in both groups and 57.5% and 61.9% of the patients with de novo and sAML, were male (*p* = 0.21), respectively.

**Table 1:** Patient, disease, and transplant characteristics

	Overall (n = 1711)	de novo (n = 1480)	sAML (n = 231)	*P*
Median follow-up (months) [quartiles]	25.9 [24.5–28.1]	26.3 [24.5–28.8]	24.6 [19.6–31.2]	0.52
Patient age (years), median (min–max) [IQR]	56.4 (18.1–82.5) [44.9–64.4]	55.8 (18.1–82.5) [44–63.8]	60.8 (20.8–75.7) [51.6–67.2]	< 0.0001
Year transplant, median (min–max)	2019 (2010–2021) [2017–2020]	2019 (2010–2021)	2019 (2010–2021)	0.58
*Cytogenetics*				
Interm	1192 (69.7%)	1037 (70.1%)	155 (67.1%)	0.36
Adverse	519 (30.3%)	443 (29.9%)	76 (32.9%)	
Time diagnosis to HSCT (mo), median (min–max) [IQR]	5.1 (1–23.9) [4–6.7]	5.2 (1–23.9) [4.1–6.7]	4.9 (1.3–20.5) [3.5–6.5]	0.005
*MRD pre transplant*				
neg	533 (63.2%)	495 (63.5%)	38 (59.4%)	0.51
pos	310 (36.8%)	284 (36.5%)	26 (40.6%)	
Missing	868	701	167	
*HT-CI*				
HT-CI = 0	798 (55.2%)	721 (57.1%)	77 (41.8%)	< 0.0001
HT-CI = 1 or 2	304 (21%)	272 (21.6%)	32 (17.4%)	
HT-CI ≥ 3	344 (23.8%)	269 (21.3%)	75 (40.8%)	
Missing	265	218	47	
*Karnofsky score*				
< 90	381 (23.3%)	311 (22%)	70 (31.5%)	0.002
≥ 90	1256 (76.7%)	1104 (78%)	152 (68.5%)	
Missing	74	65	9	
*Patient sex*				
Male	994 (58.1%)	851 (57.5%)	143 (61.9%)	0.21
Female	717 (41.9%)	629 (42.5%)	88 (38.1%)	
*Donor sex*				
Male	1052 (61.7%)	912 (61.8%)	140 (60.9%)	0.79
Female	654 (38.3%)	564 (38.2%)	90 (39.1%)	
Missing	5	4	1	
*Female to male combination*				
No F → M	1357 (79.4%)	1180 (79.8%)	177 (76.6%)	0.26
F → M	352 (20.6%)	298 (20.2%)	54 (23.4%)	
Missing	2	2	0	
*Patient CMV*				
Neg	367 (21.7%)	313 (21.3%)	54 (23.8%)	0.4
Pos	1328 (78.3%)	1155 (78.7%)	173 (76.2%)	
Missing	16	12	4	
*Donor CMV*				
neg	643 (38.1%)	545 (37.3%)	98 (43.8%)	0.063
pos	1043 (61.9%)	917 (62.7%)	126 (56.2%)	
Missing	25	18	7	
*Conditioning*				
MAC	823 (48.1%)	742 (50.1%)	81 (35.1%)	< 0.0001
RIC	888 (51.9%)	738 (49.9%)	150 (64.9%)	
*Cell source*				
BM	483 (28.2%)	433 (29.3%)	50 (21.6%)	0.017
PB	1228 (71.8%)	1047 (70.7%)	181 (78.4%)	

*sAML* secondary acute myeloid leukemia, *min* minimum, *max* maximum, *IQR* interquartile range, *Interm* intermediate, *MRD* measurable residual disease, *F* female, *M* male, *CMV *cytomegalovirus, *neg* negative, *pos* positive, *HCT CI* hematopoietic cell transplantation specific comorbidity index, *BM* bone marrow, *PB* peripheral blood, *Mac* myeloablative conditioning, *RIC* reduced intensity conditioning

In 64% of sAML patients, the antecedent hematological disorder was myelodysplastic syndrome/myeloproliferative neoplasm (MDS/MPN), while in 16.7% it was another hematological disorder, followed by solid tumor in 17% and nonmalignant hematological disorder in 2.3%. Cytogenetic risk was categorized as intermediate (70.1% vs 67.1%) or adverse (29.9% vs 32.9%) for patients with de novo AML and sAML, respectively (*p* = 0.36). Karnofsky performance status (KPS) was higher in the de novo AML group in comparison with the sAML group, with KPS ≥ 90 in 78% versus 68.5%, respectively (*p* = 0.002). HCT-CI was higher in the sAML group in comparison with the de novo AML group, with HCT-CI ≥ 3 in 40.8% versus 21.3%, respectively (*p* < 0.0001) (data were missing for 167 and 701 of the patients, respectively). There was no difference in the frequency of CMV seropositivity between the two patient groups (78.7% and 76.2%), or between the donor types (62.7% and 56.2%), respectively. Female donors to male patients were used in 23.4% and 20.2% of the cases with sAML and de novo AML, respectively (*p* = 0.26). Time from diagnosis to HaploSCT was longer in patients with de novo AML versus those with sAML; median 5.2 (range 1–23.9) versus 4.9 (range 1.3–20.5) months, respectively (*p* = 0.005). Fewer sAML patients received MAC compared to de novo AML patients, 35.1% versus 50.1%, respectively (*p* < 0.001). The most frequent conditioning regimen for both groups was thiotepa/busulfan/fludarabine at 50.2% and 45.9%, followed by busulfan/fludarabine in 17.6% and 16.5%, and fludarabine/low dose TBI in 16.2% and 19.9% of patients with de novo and sAML, respectively (Additional file 1: Table S1). Graft source was mainly peripheral blood stem cells in both de novo (70.7%) and sAML (78.4%) groups. In 53.9% and 53.7% of the de novo and AML patients, respectively, PTCY was combined with cyclosporine A (CSA) and mycophenolate mofetil (MMF), while in 30% and 27.7% it was combined with MMF and tacrolimus (Tacro), respectively (Additional file 1: Table S2).

### Transplantation outcome

Engraftment and GVHD incidence did not differ between the sAML versus de novo AML groups as depicted in Table 2. Neutrophil recovery (ANC > 0.5 × 10^9^/L) was achieved in 95.1% and 94.4% of the patients with de novo and sAML, respectively (*p* = 0.69). On day + 180, the incidence of aGVHD grades II-IV and III-IV was 27.2% (24.9–29.6%) versus 29.1% (23.1–35.3%) (*p* = 0.55) and 9.2% (7.7–10.8%) versus 6.6% (3.8–10.4%), respectively (*p* = 0.23). Two-year incidence of total and extensive cGVHD was 32.2% (29.5–34.9%) versus 33.3% (26.5–40.3%) (*p* = 1) and 11.9% (10.1–13.9%) versus 11.3% (7.1–16.6%), respectively (*p* = 0.48). Similarly, two-year NRM and RI as well as LFS, OS, and GRFS did not differ between the sAML versus de novo AML groups. Two-year NRM and RI were 21.1% (18.29–23.4%) versus 20.8% (15.4–26.7%) (*p* = 0.72) and 19.5% (17.2–21.8%) versus 21.3% (15.6–27.5%) (*p* = 0.39) in de novo versus sAML, respectively (Table 3A). The 2-year LFS, OS, and GRFS were 59.5% (56.6–62.2%) versus 58% (50.5–64.7%) (*p* = 0.28), 65.4% (62.5–68%) versus 66.7% (58.3–72.1%) (*p* = 0.35) and 49.9% (47–52.7%) versus 47% (39.5–54.1%) (*p* = 0.5), respectively (Table 3A). Also no difference was observed in any transplantation outcome parameter between sAML post MDS/MPN/ bone marrow failure syndrome (BMFS) versus de novo AML and sAML post other malignant hematological disorders (OMHD) /solid tumor (ST) versus de novo AML (Table 3B).

**Table 2:** Transplantation outcomes: engraftment and acute graft versus host disease

	Overall (n = 1711)	de novo (n = 1480)	sAML (n = 231)	*P*
*Engraftment HSCT*				
Graft failure	82 (5%)	70 (4.9%)	12 (5.6%)	0.69
Engrafted	1558 (95%)	1354 (95.1%)	204 (94.4%)	
Mssing	71	56	15	
cumulative incidence of PMN > 500, day 30		91.1% [89.5–92.4]	86.6% [81.2–90.5]	0.037
*Acute GVHD*				
Grade I	285 (17.4%)	249 (17.5%)	36 (16.7%)	Not done
Grade II	303 (18.5%)	255 (18%)	48 (22.3%)	
Grade III	103 (6.3%)	93 (6.5%)	10 (4.7%)	
Grade IV	38 (2.3%)	34 (2.4%)	4 (1.9%)	
Present, grade unknown	23 (1.4%)	21 (1.5%)	2 (0.9%)	
No aGvHD present (Grade 0)	883 (54%)	768 (54.1%)	115 (53.5%)	
Missing	76	60	16	

*sAML* secondary acute myeloid leukemia, *HSCT* hematopoietic stem cell transplantation, *GVHD* graft-versus-host disease, *a* acute, *PMN* polymorphonuclear cells

**Table 3:** (A) transplantation outcomes, (B) transplantation outcomes: sAML per antecedent hematological disorder versus de novo AML

(A)					
	2 years				
	Relapse	NRM	LFS	OS	GRFS
de novo	19.5% [17.2–21.8]	21.1% [18.9–23.4]	59.5% [56.6–62.2]	65.4% [62.5–68]	49.9% [47–52.7]
sAML	21.3% [15.6–27.5]	20.8% [15.4–26.7]	58% [50.5–64.7]	65.7% [58.3–72.1]	47% [39.5–54.1]
P value	0.39	0.72	0.28	0.35	0.5

	180 days		2 years	
	Acute GVHD II-IV	Acute GVHD III-IV	chronic GVHD	ext. chronic GVHD
de novo	27.2% [24.9–29.6]	9.2% [7.7–10.8]	32.2% [29.5–34.9]	11.9% [10.1–13.9]
secAML	29.1% [23.1–35.3]	6.6% [3.8–10.4]	33.3% [26.5–40.3]	11.3% [7.1–16.6]
*P* value	0.55	0.23	1	0.48

(B)					
	2 years				
	Relapse	NRM	LFS	OS	GRFS
de novo	19.5% [17.2–21.8]	21.1% [18.9–23.4]	59.5% [56.6–62.2]	65.4% [62.5–68]	49.9% [47–52.7]
MDS/MPN/BMFS	21.6% [14.6–29.6]	17.3% [11.3–24.2]	61.1% [51.7–69.2]	70.6% [61.5–77.8]	46.9% [37.4–55.8]
OMHD/ST	20.7% [11.9–31.2]	27.1% [17.2–38.1]	52.2% [39.6–63.4]	57.1% [44.2–68.1]	46.7% [34.3–58.1]
*P* value	0.68	0.58	0.3	0.22	0.79

	180 days		2 years	
	Acute GVHD II-IV	Acute GVHD III-IV	Chronic GVHD	Ext. chronic GVHD
de novo	27.2% [24.9–29.6]	9.2% [7.7–10.8]	32.2% [29.5–34.9]	11.9% [10.1–13.9]
MDS/MPN/BMFS	30.9% [23.4–38.7]	6.5% [3.2–11.4]	38.2% [29.2–47.1]	13.7% [7.9–21.1]
OMHD/ST	25.7% [16.3–36.1]	6.8% [2.5–14]	24.4% [14.7–35.4]	7.4% [2.7–15.3]
*P* value	0.59	0.49	0.19	0.36

*sAML* secondary acute myeloid leukemia, *NRM* non-relapse mortality, *LFS* leukemia-free survival, *OS* overall survival, *GVHD* graft-versus-host disease, *GRFS* GVHD-free, relapse-free survival, *ext* extensive, *MDS/MPN/BMF* sAML post myelodysplastic syndrome, myeloproliferative neoplasm, bone marrow failure syndrome, *OMHD/ST* sAML post other malignant hematologic disorders and solid tumors

### Multivariate analysis

In multivariate analysis (Table 4A), no difference was observed in any transplantation outcome parameter between the sAML versus de novo AML groups; The HR for NRM was 0.87 (95% CI 0.7–1.35, *p* = 0.87), RI HR = 1.02(95% CI 0.72–1.45, *p* = 0.9), LFS HR = 1, (95% CI 0.79–1.27, *p* = 0.99), OS HR = 0.95 (95% CI 0.74–1.23, *p* = 0.72) and GRFS HR = 0.94 (95% CI 0.75–1.17, *p* = 0.57) (Table 4). Similarly, the incidence of a GVHD II-IV HR = 1.04 (95% CI 0.77–1.41, *p* = 0.8), aGVHD III-IV HR = 0.73 (95% CI 0.41–1.32, *p* = 0.3), cGVHD all grades HR = 0.96 (95% CI 0.71–1.29, *p* = 0.78) and extensive cGVHD HR = 0.65 (95% CI 0.39–1.08, *p* = 0.097) did not differ between the two groups (Table 4A). Also no difference was observed in any transplantation outcome parameter between sAML post MDS/MPN/BMFS versus de novo AML and sAML post OMHD/ST versus de novo AML (Table 4B). Other significant prognostic factors were adverse cytogenetics risk predicting higher RI and lower LFS, OS, and GRFS; age (per 10 years) predicted higher NRM and inferior LFS, OS, and GRFS. KPS > 90 was a prognostic factor for lower NRM and higher LFS, OS, and GRFS. RIC was associated with higher RI and worse LFS and OS. Female donor to male patient combination was associated was lower LFS, OS, GRFS and higher extensive cGVHD. A peripheral blood graft was a predictive factor for a higher incidence of grade II–IV and III–IV aGVHD as well as total cGVHD (Table 4).

**Table 4:** (A) Multivariate analysis, (B) multivariate analysis-sAML per antecedent hematological disorder versus de novo AML

(A)						
	RELAPSE		NRM		LFS	
	HR (95% CI)	*p* value	HR (95% CI)	*p* value	HR (95% CI)	*p* value
sAML versus do novo	1.02 (0.72–1.45)	0.91	0.97 (0.7–1.35)	0.87	1 (0.79–1.27)	0.99
Patient age (per 10 years)	0.98 (0.89–1.08)	0.62	1.36 (1.21–1.52)	< 0.0001	1.14 (1.06–1.23)	5.00E−04
Adverse versus intermediate cytogenetics	1.95 (1.53–2.49)	< 0.0001	1.22 (0.95–1.56)	0.12	1.54 (1.29–1.83)	< 0.0001
Time diagnosis to HSCT (mo)	1 (0.96–1.04)	0.86	1.03 (0.99–1.06)	0.13	1.01 (0.99–1.04)	0.36
KPS > 90	0.79 (0.59–1.04)	0.09	0.7 (0.54–0.92)	0.009	0.73 (0.6–0.88)	0.001
RIC versus MAC	1.4 (1.06–1.85)	0.016	1.1 (0.84–1.45)	0.48	1.24 (1.02–1.5)	0.031
Female to male versus other	1.22 (0.91–1.63)	0.18	1.25 (0.95–1.65)	0.1	1.24 (1.02–1.52)	0.031
Pat. CMV pos	1.04 (0.77–1.42)	0.78	1.33 (0.95–1.85)	0.094	1.17 (0.93–1.46)	0.18
Don. CMV pos	0.85 (0.65–1.1)	0.21	1.14 (0.88–1.48)	0.32	0.99 (0.82–1.19)	0.93
PBSC versus BM	0.91 (0.7–1.19)	0.49	0.93 (0.7–1.22)	0.58	0.92 (0.76–1.11)	0.39
Centre (frailty term)		0.24		0.014		0.11

	OS		GRFS		Acute GVHD II-IV	
	HR (95% CI)	*p* value	HR (95% CI)	*p* value	HR (95% CI)	*p* value
sAML versus do novo	0.95 (0.74–1.23)	0.72	0.94 (0.75–1.17)	0.57	1.04 (0.77–1.41)	0.8
Patient age (per 10 years)	1.22 (1.13–1.33)	< 0.0001	1.09 (1.02–1.17)	0.008	1.07 (0.97–1.17)	0.16
Adverse versus intermediate cytogenetics	1.56 (1.29–1.87)	< 0.0001	1.44 (1.24–1.69)	< 0.0001	0.99 (0.8–1.24)	0.94
Time diagnosis to HSCT (mo)	1.01 (0.99–1.04)	0.34	1 (0.98–1.03)	0.71	0.97 (0.94–1.01)	0.098
KPS > 90	0.74 (0.6–0.91)	0.005	0.81 (0.68–0.97)	0.019	0.82 (0.64–1.06)	0.13
RIC versus MAC	1.29 (1.05–1.59)	0.018	1.18 (0.99–1.41)	0.072	0.95 (0.74–1.22)	0.68
Female to male versus other	1.3 (1.05–1.6)	0.015	1.28 (1.07–1.53)	0.006	0.81 (0.62–1.05)	0.12
Pat. CMV pos	1.16 (0.91–1.48)	0.23	1.1 (0.9–1.34)	0.35	1.01 (0.77–1.31)	0.97
Don. CMV pos	1.05 (0.86–1.28)	0.65	0.99 (0.84–1.18)	0.95	1.02 (0.81–1.27)	0.89
PBCS versus BM	0.89 (0.72–1.09)	0.26	1.12 (0.93–1.34)	0.23	1.79 (1.35–2.39)	< 0.0001
Centre (frailty term)		0.017		0.015		< 0.0001

	Acute GVHD III–IV		Chronic GVHD		Extensive chronic GVHD	
	HR (95% CI)	*p* value	HR (95% CI)	*p* value	HR (95% CI)	*p* value
sAML versus do novo	0.73 (0.41–1.32)	0.3	0.96 (0.71–1.29)	0.78	0.65 (0.39–1.08)	0.097
Patient age (per 10 years)	1.01 (0.87–1.17)	0.92	1.07 (0.99–1.17)	0.1	1.09 (0.95–1.25)	0.24
Adverse versus intermediate cytogenetics	1.03 (0.7–1.51)	0.89	1.06 (0.85–1.32)	0.63	1.32 (0.93–1.86)	0.11
Time diagnosis to HSCT (mo)	0.98 (0.92–1.04)	0.5	1.01 (0.97–1.04)	0.76	1 (0.94–1.05)	0.88
KPS > 90	0.8 (0.52–1.21)	0.29	1.06 (0.82–1.37)	0.68	1.05 (0.7–1.58)	0.82
RIC versus MAC	0.93 (0.61–1.39)	0.71	1.14 (0.9–1.45)	0.27	1.11 (0.76–1.64)	0.58
Female to male versus other	0.89 (0.56–1.41)	0.63	1.17 (0.92–1.49)	0.2	1.52 (1.05–2.19)	0.027
Pat. CMV pos	0.9 (0.58–1.41)	0.66	0.97 (0.74–1.26)	0.81	0.99 (0.64–1.54)	0.97
Don. CMV pos	1.01 (0.68–1.49)	0.98	1.02 (0.81–1.27)	0.89	1.21 (0.83–1.76)	0.31
PBCS versus BM	1.73 (1.09–2.74)	0.019	1.55 (1.19–2.02)	0.001	1.52 (0.99–2.34)	0.056
Centre (frailty term)		0.24		0.0005		0.0001

(B)						
	RELAPSE		NRM		LFS	
	HR (95% CI)	*p* value	HR (95% CI)	*p* value	HR (95% CI)	*p* value
de novo AML (reference)	1		1		1	
MDS/MPN/BMFS	1.02 (0.67–1.56)	0.91	0.87 (0.57–1.31)	0.5	0.95 (0.71–1.27)	0.73
OMHD/ST	1.01 (0.58–1.76)	0.96	1.18 (0.72–1.93)	0.52	1.1 (0.76–1.59)	0.62
Patient age (per 10 years)	0.98 (0.89–1.08)	0.62	1.36 (1.21–1.52)	< 0.0001	1.14 (1.06–1.23)	0.0005
Adverse versus intermediate cytogenetics	1.95 (1.53–2.49)	< 0.0001	1.21 (0.94–1.56)	0.13	1.53 (1.29–1.82)	< 0.0001
Time diagnosis to HSCT (mo)	1 (0.96–1.04)	0.86	1.03 (0.99–1.06)	0.14	1.01 (0.99–1.04)	0.38
KPS > 90	0.79 (0.59–1.04)	0.09	0.7 (0.54–0.92)	0.009	0.73 (0.6–0.89)	0.001
RIC versus MAC	1.4 (1.06–1.85)	0.016	1.1 (0.84–1.45)	0.48	1.24 (1.02–1.5)	0.031
Female to male versus other	1.22 (0.91–1.63)	0.18	1.27 (0.96–1.67)	0.091	1.25 (1.02–1.52)	0.029
Pat. CMV pos	1.04 (0.77–1.42)	0.78	1.32 (0.95–1.85)	0.098	1.17 (0.93–1.46)	0.18
Don. CMV pos	0.85 (0.65–1.1)	0.21	1.14 (0.88–1.48)	0.32	0.99 (0.82–1.19)	0.93
PBSC versus BM	0.91 (0.7–1.19)	0.49	0.92 (0.7–1.22)	0.57	0.92 (0.76–1.11)	0.38
Centre (frailty term)		0.24		0.012		0.1

	OS		GRFS		Acute GVHD II–IV	
	HR (95% CI)	*p* value	HR (95% CI)	*p* value	HR (95% CI)	*p* value
de novo AML (reference)	1		1		1	
MDS/MPN/BMFS	0.88 (0.64–1.21)	0.43	0.97 (0.75–1.25)	0.8	1.09 (0.76–1.55)	0.65
OMHD/ST	1.1 (0.74–1.63)	0.63	0.89 (0.62–1.27)	0.52	0.95 (0.57–1.58)	0.84
Patient age (per 10 years)	1.22 (1.13–1.33)	< 0.0001	1.09 (1.02–1.17)	0.009	1.07 (0.97–1.17)	0.16
Adverse versus intermediate cytogenetics	1.55 (1.29–1.87)	< 0.0001	1.45 (1.24–1.69)	< 0.0001	0.99 (0.8–1.24)	0.96
Time diagnosis to HSCT (mo)	1.01 (0.98–1.04)	0.36	1 (0.98–1.03)	0.69	0.97 (0.94–1.01)	0.1
KPS > 90	0.74 (0.6–0.91)	0.005	0.81 (0.67–0.96)	0.019	0.82 (0.64–1.05)	0.12
RIC versus MAC	1.29 (1.04–1.59)	0.018	1.18 (0.99–1.41)	0.071	0.95 (0.74–1.22)	0.68
Female to male versus other	1.3 (1.06–1.61)	0.014	1.28 (1.07–1.53)	0.007	0.81 (0.62–1.05)	0.11
Pat. CMV pos	1.16 (0.91–1.47)	0.24	1.1 (0.9–1.34)	0.35	1.01 (0.77–1.31)	0.97
Don. CMV pos	1.05 (0.86–1.28)	0.66	0.99 (0.84–1.18)	0.95	1.02 (0.81–1.27)	0.9
PBCS versus BM	0.89 (0.72–1.09)	0.25	1.12 (0.93–1.34)	0.23	1.79 (1.35–2.39)	< 0.0001
Centre (frailty term)		0.015		0.016		< 0.0001

	Acute GVHD III-IV		Chronic GVHD		Extensive chronic GVHD	
	HR (95% CI)	*p* value	HR (95% CI)	*p* value	HR (95% CI)	*p* value
de novo AML (reference)	1		1		1	
MDS/MPN/BMFS	0.77 (0.39–1.55)	0.47	1.14 (0.82–1.6)	0.42	0.81 (0.46–1.42)	0.46
OMHD/ST	0.66 (0.24–1.81)	0.42	0.62 (0.35–1.09)	0.099	0.32 (0.1–1.02)	0.055
Patient age (per 10 years)	1.01 (0.87–1.17)	0.92	1.07 (0.99–1.17)	0.099	1.08 (0.94–1.24)	0.25
Adverse versus intermediate cytogenetics	1.03 (0.7–1.51)	0.88	1.07 (0.86–1.33)	0.56	1.34 (0.95–1.89)	0.098
Time diagnosis to HSCT (mo)	0.98 (0.92–1.04)	0.51	1.01 (0.98–1.04)	0.66	1 (0.95–1.05)	0.97
KPS > 90	0.8 (0.52–1.21)	0.29	1.05 (0.81–1.36)	0.7	1.03 (0.69–1.55)	0.88
RIC versus MAC	0.93 (0.61–1.4)	0.71	1.14 (0.9–1.45)	0.27	1.12 (0.76–1.65)	0.55
Female to male versus other	0.89 (0.56–1.41)	0.62	1.17 (0.92–1.49)	0.21	1.52 (1.05–2.2)	0.027
Pat. CMV pos	0.91 (0.58–1.41)	0.66	0.97 (0.75–1.27)	0.83	0.99 (0.64–1.54)	0.98
Don. CMV pos	1.01 (0.68–1.49)	0.98	1.02 (0.81–1.27)	0.88	1.22 (0.84–1.77)	0.3
PBCS versus BM	1.73 (1.09–2.74)	0.019	1.55 (1.19–2.02)	0.001	1.52 (0.99–2.34)	0.055
Centre (fraitly term)		0.24		0.0006		0.0002

*sAML* secondary acute myeloid leukemia, *MDS/MPN/BMF* sAML post myelodysplastic syndrome, myeloproliferative neoplasm, bone marrow failure syndrome, *OMHD/ST* sAML post other malignant hematologic disorders and solid tumors, *HSCT* hematopoietic stem cell transplantation, *mo* month, *NRM* non-relapse mortality, *RI* relapse incidence, *LFS* leukemia-free survival, *OS* overall survival, *GVHD* graft-versus-host disease, *GRFS* GVHD-free, relapse-free survival, *pos* positive, *KPS* Karnofsky performance score, *MAC* myeloablative conditioning, *RIC* reduced intensity conditioning, *CMV* cytomegalovirus, *Pat.* patient, *Don.* Donor, *BM* bone marrow, *PBSC* peripheral blood stem cells

### Cause of death

A total of 485 (32.8%) patients with de novo AML and 79 (34.2%) with sAML died during the study period (Table 5). The original disease was the main cause of death accounting for 36.3% and 42.1% of the deaths, respectively. The second cause of death was infection at 30.5% and 26.3%, followed by GVHD with 14.6% and 9.2% of deaths, in patients with de novo and sAML, respectively (Table 5). Multi-organ failure accounted for 2.4% and 3.9% of the deaths, respectively. Other causes of death were infrequent and included veno-occlusive disease of the liver, cardiac toxicity, hemorrhage, graft failure, and central nervous system toxicity, each accounting for less than 2% of total deaths with no difference between the patient groups (Table 5).

**Table 5:** Cause of death

	Overall (n = 564)	de novo (n = 485)	sAML (n = 79)
Original disease	201 (37.2%)	169 (36.3%)	32 (42.1%)
Infection	162 (29.9%)	142 (30.5%)	20 (26.3%)
GVHD	75 (13.9%)	68 (14.6%)	7 (9.2%)
Non HSCT related	34 (6.3%)	26 (5.6%)	8 (10.5%)
Other transp related	17 (3.1%)	17 (3.7%)	0 (0%)
MOF	14 (2.6%)	11 (2.4%)	3 (3.9%)
VOD	10 (1.8%)	10 (2.2%)	0 (0%)
Cardiac toxicity	7 (1.3%)	6 (1.3%)	1 (1.3%)
Other second malignancy	6 (1.1%)	5 (1.1%)	1 (1.3%)
Haemorhage	6 (1.1%)	4 (0.9%)	2 (2.6%)
Failure/Rejection	4 (0.7%)	3 (0.6%)	1 (1.3%)
CNS toxicity	4 (0.7%)	3 (0.6%)	1 (1.3%)
IP	1 (0.2%)	1 (0.2%)	0 (0%)
Missing	23	20	3

*sAML* secondary acute myeloid leukemia, *GVHD* graft-versus-host disease, *HSCT* hematopoietic stem cell transplantation, *MOF* multi organ failure, *VOD* veno-occlusive disease of the liver, *CNS* central nervous system, *IP* interstitial pneumonitis

### Matched-pair analysis

**Fig. 1:**
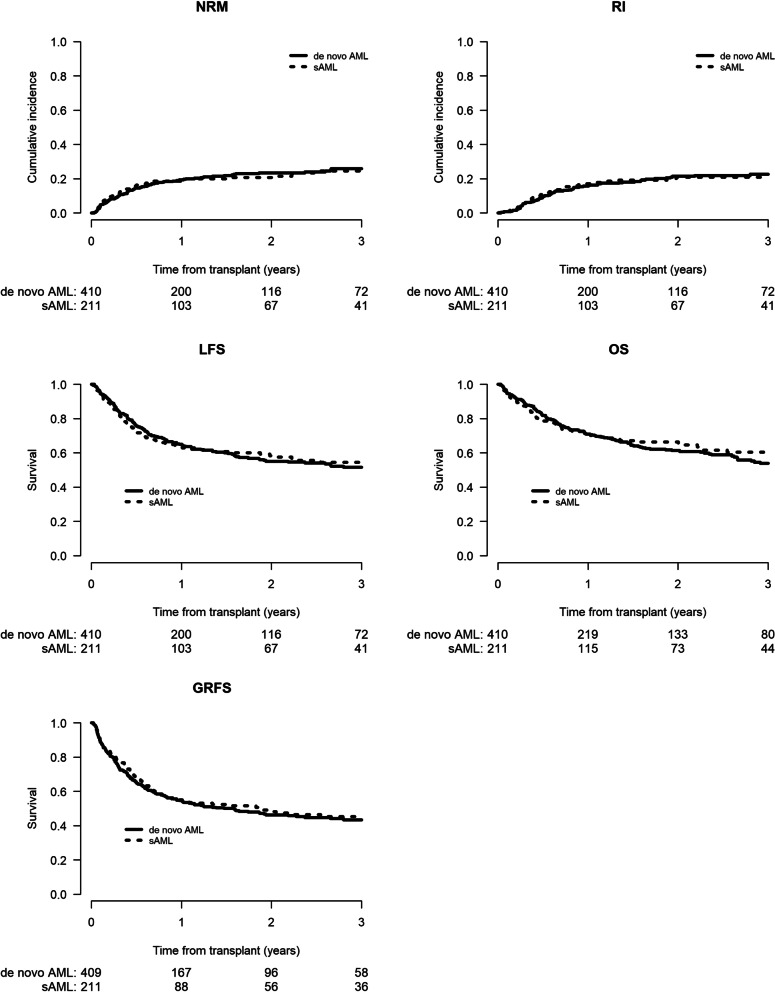
Matched-pair analysis of haploidentical transplantation outcomes in secondary AML versus de novo AML: non-relapse mortality (NRM), relapse incidence (RI), leukemia-free survival (LFS), overall survival (OS), and GVHD-free, relapse-free survival (GRFS)

**Fig. 2:**
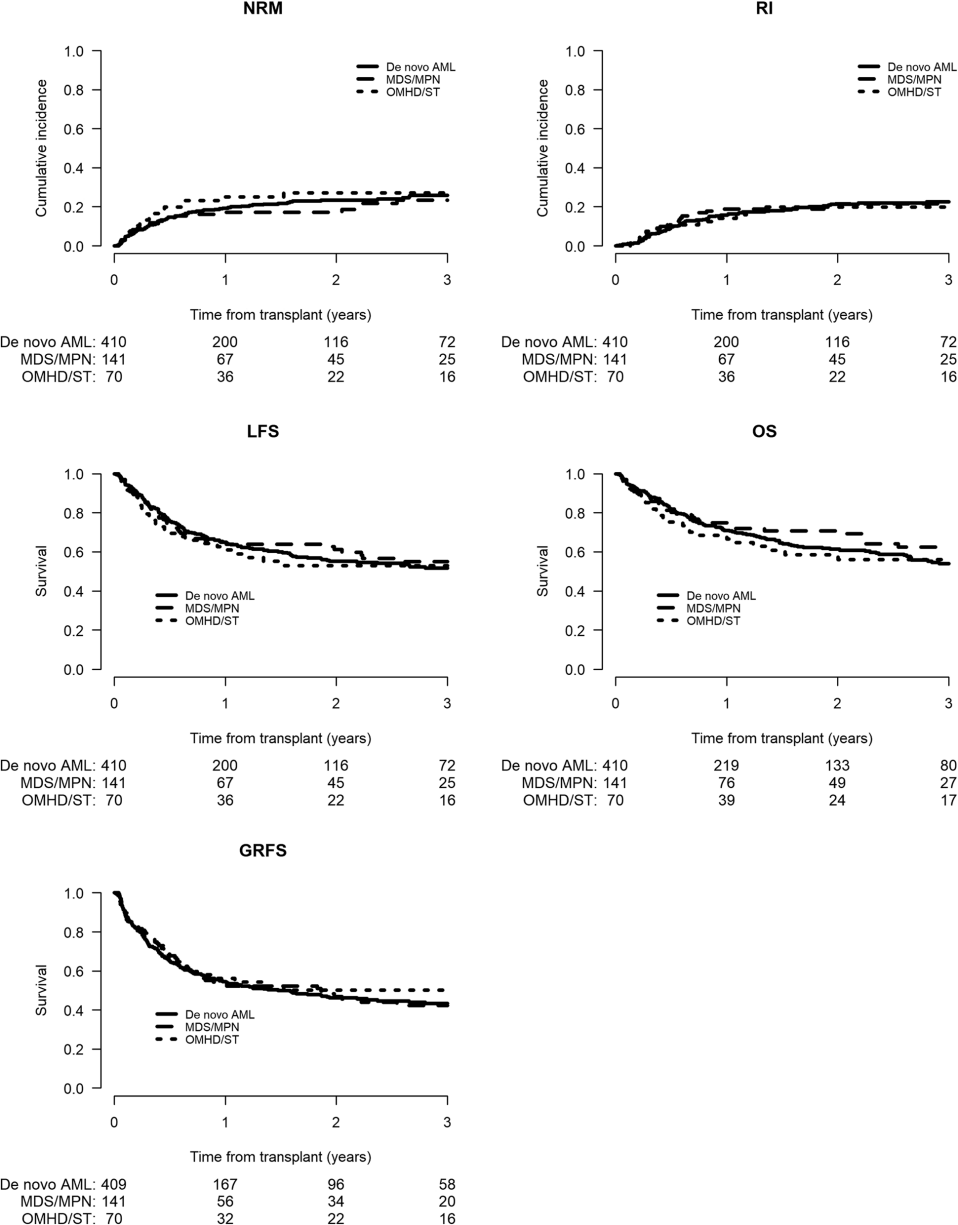
Matched-pair analysis of haploidentical transplantation outcomes in secondary AML per antecedent hematological disorder versus de novo AML: non-relapse mortality (NRM), relapse incidence (RI), leukemia-free survival (LFS), overall survival (OS), and GVHD-free, relapse-free survival (GRFS). sAML post myelodysplastic syndrome (MDS), myeloproliferative neoplasm (MPN), bone marrow failure syndrome (BMF) (MDS /MPN) (n-141); sAML post other malignant hematologic disorders(OMDS), solid tumors (ST) (OMDS/ST) (n-70)

To minimize the effect of confounding factors, a matched-pair analysis (2:1 ratio) was performed. Using the criteria mentioned above, 621 well-matched pairs (de novo AML = 410; sAML = 211 were identified (Additional file 1: Tables S3–S7). In 141 of the sAML patients the antecedent hematological disorder was MDS or MPN or bone marrow failure syndrome (BMFS) while in 70 patients the antecedent disease was other malignant haematological disorder (OMHD) [10] or solid tumor (ST), respectively. The results of the matched-pair analysis were consistent with previous results for the entire population. Engraftment was 93.4% versus 94.9% in de novo and sAML, respectively (*p* = 0.47) (Additional file 1: Table S4). Incidence of both acute and cGVHD was similar between the 2 cohorts: aGVHD Grade II-IV 27.6% (23.2–32.1) versus 27.7% (21.6–34.1), HR = 0.99 (95% CI 0.72–1.38, *p* = 0.96), aGVHD Grade III-IV 9.5% (6.9–12.7) versus 6.7% (3.7–10.8), HR = 0.7 (95% CI 0.36–1.35, *p* = 0.29), total cGVHD 31.8% (26.7–37%) versus 32.8% (25.7–40.1%), HR = 1.03 (95% CI 0.75–1.42, *p* = 0.84) and extensive cGVHD 10.2% (7.2–13.8%) versus 10.6% (6.4–16.1%), HR = 0.93 (95% CI 0.53–1.61, *p* = 0.79), respectively (Table 6A). Two-year NRM and RI did not differ with HaploSCT for de novo versus sAML; 23.4% (19–28.1) versus 20.6% (15.1–26.8%), HR = 0.92(95% CI 0.64–1.33, *p* = 0.67) and 21.4% (17–26.1%) versus 21% (15.1–27.5%), HR = 0.98 (95% CI 0.672–1.42, *p* = 0.9), respectively (Table 6A). There was also no difference in LFS, OS, and GRFS between the de novo AML and sAML groups 55.2% (49.5–60.5%) versus 58.4% (50.6–65.4%), HR = 0.95 (95% CI 0.74–1.22, *p* = 0.67); 61.4% (55.7–66.5%) versus 66.4% (58.8–73%), HR = 0.91 (95% CI 0.69–1.2, *p* = 0.51) and 46.3% (40.7–51.6%) versus 48.2% (40.4–55.6%), HR = 0.92 (95% CI 0.73–1.16, *p* = 0.48), respectively (Table 6A, Fig. 1). No difference was observed in any transplantation outcome parameter between sAML post MDS/MPN/BMFS versus de novo AML and sAML post OMHD/ST versus de novo AML (Table 6B, Fig. 2). Finally, we also verified that the results are consistent when adjusting the comparison on HCT-CI (data not shown). Causes of death are listed in Additional file 1: Table S7.

**Table 6:** (A) Matched-pair analysis, (B) matched-pair analysis: results; sAML per antecedent hematological disorder versus de novo AML

(A)					
	2 years				
	Relapse	NRM	LFS	OS	GRFS
de novo	21.4% [17–26.1]	23.4% [19–28.1]	55.2% [49.5–60.5]	61.4% [55.7–66.5]	46.3% [40.7–51.6]
sAML	21% [15.1–27.5]	20.6% [15.1–26.8]	58.4% [50.6–65.4]	66.4% [58.8–73]	48.2% [40.4–55.6]
sAML versus de novo AML	0.98 (0.67–1.42)	0.92 (0.64–1.33)	0.95 (0.74–1.22)	0.91 (0.69–1.2)	0.92 (0.73–1.16)
p value (cluster = pair)	0.9	0.67	0.67	0.51	0.48

	180 days		2 years	
	Acute GVHD II-IV	Acute GVHD III-IV	chronic GVHD	ext. chronic GVHD
de novo	27.6% [23.2–32.1]	9.5% [6.9–12.7]	31.8% [26.7–37]	10.2% [7.2–13.8]
sAML	27.7% [21.6–34.1]	6.7% [3.7–10.8]	32.8% [25.7–40.1]	10.6% [6.4–16.1]
sAML versus de novo AML	0.99 (0.72–1.38)	0.7 (0.36–1.35)	1.03 (0.75–1.42)	0.93 (0.53–1.61)
*p* value (cluster = pair)	0.96	0.29	0.84	0.79

(B)					
	2 years				
	Relapse	NRM	LFS	OS	GRFS
de novo AML	21.4% [17–26.1]	23.4% [19–28.1]	55.2% [49.5–60.5]	61.4% [55.7–66.5]	46.3% [40.7–51.6]
MDS/MPN/BMFS	21.5% [14.3–29.7]	17.2% [11–24.5]	61.3% [51.5–69.7]	70.7% [61.4–78.2]	47% [37.2–56.2]
OMHD/ST	19.9% [10.8–30.9]	27.1% [16.6–38.7]	53% [39.6–64.8]	58.6% [44.8–70.1]	50.1% [36.8–62]
*MDS/MPN/BMFS versus de novo*					
HR (95% CI)	0.98 (0.63–1.52)	0.84 (0.53–1.31)	0.9 (0.67–1.22)	0.84 (0.6–1.18)	0.96 (0.74–1.25)
p value	0.92	0.44	0.5	0.33	0.76
*OMHD/ST*					
HR (95% CI)	0.98 (0.55–1.75)	1.09 (0.64–1.84)	1.04 (0.7–1.53)	1.04 (0.69–1.58)	0.85 (0.58–1.24)
p value	0.94	0.75	0.85	0.85	0.4

	180 days		2 years	
	Acute GVHD II–IV	Acute GVHD III–IV	Chronic GVHD	ext. chronic GVHD
de novo AML	27.6% [23.2–32.1]	9.5% [6.9–12.7]	31.8% [26.7–37]	10.2% [7.2–13.8]
MDS/MPN/BMFS	29.1% [21.5–37.2]	7.1% [3.5–12.4]	38.3% [29–47.5]	13.8% [7.8–21.4]
OMHD/ST	25% [15.4–35.8]	5.9% [1.9–13.3]	22.3% [12.5–33.9]	5% [1.3–12.8]
*MDS/MPN/BMFS versus de novo*				
HR (95% CI)	1.05 (0.73–1.51)	0.75 (0.35–1.57)	1.24 (0.88–1.74)	1.22 (0.68–2.17)
p value	0.78	0.44	0.22	0.5
*OMHD/ST*				
HR (95% CI)	0.88 (0.52–1.49)	0.62 (0.21–1.78)	0.67 (0.38–1.19)	0.42 (0.13–1.39)
*p* value	0.64	0.37	0.17	0.15

*sAML* secondary acute myeloid leukemia, *MDS/MPN/BMF* sAML post myelodysplastic syndrome, myeloproliferative neoplasm, bone marrow failure syndrome, *OMHD/ST* sAML post other malignant hematologic disorders and solid tumors, *NRM* non-relapse mortality, *LFS* leukemia-free survival, *OS* overall survival, *GVHD* graft-versus-host disease, *GRFS* GVHD-free, relapse-free survival, *ext* extensive

## Discussion

In this study, we have demonstrated similar transplantation outcomes for patients with sAML in comparison to those with de novo AML following non-T depleted HaploSCT and PTCy. Furthermore, no difference was observed in transplantation outcomes irrespectively of the antecedent hematological disorder. Impressively, about two thirds of the sAML patients were rescued and half of them were relapse-free and GVHD free at 2 years. These results are similar and even slightly better than the results we previously published on behalf of the ALWP of the EBMT analyzing transplantation outcomes in 154 sAML patients undergoing non-T depleted HaploSCT between 2006 to 2016, 119 of them with PTCy, and a 2-year LFS, OS, and GRFS of 37.1%, 43.3% and 42.1%, respectively [17]. In a subsequent study that included 246 HaploSCT performed in a similar period, 2-year LFS, OS, and GRFS were 32%, 41%, and 23%, respectively [18]. Schmaelter et al. from the ALWP of the EBMT compared transplantation results in 11,439 patients with de novo and 1325 with sAML (8600 of whom were in CR1) transplanted mostly from sibling and unrelated donors and observed a higher RI and also higher NRM in sAML versus de novo AML, which translated to significantly inferior LFS, OS, and GRFS in the sAML patients with HRs of 1.33, 1.32 and 1.2, respectively [10]. Historically, conventional therapeutic results in sAML are inferior to those in de novo AML due to multiple reasons including more aggressive disease biology, more unfavorable cytogenetics and mutation rates, the antecedent malignancy, and previous therapies upregulating multidrug resistance genes, and thus a poor response to chemotherapy as well as patient-related factors such as older age, and comorbidities leading to reduced tolerability to chemotherapy with increased toxicity and side effects [3–7, 21, 31–33]. These poor prognostic factors are also operating in the setting of transplantation resulting in both higher relapse rates as well as higher NRM which translate to inferior outcomes including LFS and OS, and GRFS in patients with sAML in comparison to those with de novo AML [8–12, 14, 34]. However, the scenario with non-T depleted HaploSCT especially with PTCy may differ due to the unique biology of the PTCy platform leading to a remarkable reduction in transplant-related mortality and GVHD, translating into improved results [15, 16, 35–37]. Furthermore, the Haplo procedure may be associated with enhanced anti-leukemic efficacy as was recently nicely proved by Professor Huang Xia June in a mice model which carried the human AML-ETO or MLL-AF9 fusion gene showing that cytotoxic T lymphocytes from the haploSCT group had higher cytotoxicity than those from the MSD group [38]. Although controversial, the GVL effect may be stronger with non-T cell-depleted Haplo donors with faster clearance of post-transplant measurable residual disease, reduced post-transplant disease progression, and relapse, and better results in positive MRD pre-transplantation high-risk leukemia as compared to sibling transplantation [19, 20, 38–42]. Furthermore, it is conceivable that the GVL effect is not the only mechanism that protects from disease relapse when using PTCy. The PTCy strategy may provide a direct immune-mediated specific anti-leukemic effect, distinct from GVHD, that is probably mediated by the release of cytokines or other molecules to which leukemic cells may be more sensitive than normal cells [43]. Altogether the reduced toxicity and potentially stronger anti-leukemic effect may be of special importance in patients with sAML and may explain the lack of difference we observed with the Haplo transplants in patients with sAML versus those with de novo AML. Furthermore, our data were analyzed using a propensity score analysis in order to balance the characteristics of the two populations. The matched-pair analysis confirmed the results that we found in the standard analysis indicating similar main outcomes post-HaploSCT in sAML and de novo AML. Our data are somewhat similar and in agreement with a recent report by our Chinese colleagues that demonstrated that the prognosis of haploSCT in patients with AML with myelodysplasia related changes (AML-MRC) in first CR is similar to that of other types of high-risk AML patients and that HaploSCT is an ideal choice for patients with AML-MRC in CR [44].

As previously reported for de novo AML and MDS, we observed a lower relapse rate with MAC as compared to RIC in agreement with a previous publication where we demonstrated lower RI and better LFS and OS by including patients with sAML post-MDS and patients with AML undergoing second transplants [14, 45, 46].

The other factors observed to be associated with HaploSCT outcomes included cytogenetic risk, age, KPS, and female donor-to-male patient combination and are in agreement with previous publications of allogeneic transplantations including HaploSCTs in de novo AML [9–11, 22, 45–49].

This study, being a retrospective and registry-based transplantation study, has several limitations including the risk of selection bias and the possibility of unavailable data that could not have been considered, such as frontline therapies as well as molecular, MRD, and CD34 cell dose data. Also, we included in our analysis only patients in CR1 that are thus with favorable outcomes, and results in more advanced stages of sAML may differ, especially as sAML is typically associated with lower and shorter CRs compared to de novo AML.

In conclusion, in this relatively large registry-based retrospective analysis of HaploSCT for sAML in comparison to HaploSCT in de novo AML, we observed similar transplantation outcomes with HaploSCT being about two-thirds of the patients with this devastating leukemia. Hopefully, the recently approved novel agents (mainly vyxeos [CPX-351]) that have been shown to enable more sAML patients to undergo HLA-matched allogeneic transplantation and hopefully also HaploSCTs, it may be possible to further improve sAML outcomes [50].

### Supplementary Information

**Additional file 1**. Contributing centers and Supplemental Tables.

## Data Availability

A.N., M.N., M.L., and M.M. had full access to all study data (available upon data-specific request).
