# Correction: Non-T-depleted haploidentical transplantation with post-transplant cyclophosphamide in patients with secondary versus de novo AML in first complete remission: a study from the ALWP/EBMT

**DOI:** 10.1186/s13045-023-01501-w

**Published:** 2023-09-29

**Authors:** Arnon Nagler, Myriam Labopin, Didier Blaise, Anna Maria Raiola, Lucia Lopez Corral, Stefania Bramanti, Simona Sica, Mi Kwon, Yener Koc, Jiri Pavlu, Alexander Kulagin, Alessandro Busca, Arancha Bermúdez Rodríguez, Péter Reményi, Christoph Schmid, Eolia Brissot, Jaime Sanz, Ali Bazarbachi, Sebastian Giebel, Fabio Ciceri, Mohamad Mohty

**Affiliations:** 1 Division of Hematology, Sheba Medical Center, Tel Hashomer, Israel; 2 EBMT Paris Study Office, Department of Haematology, Saint Antoine Hospital; INSERM UMR 938, Sorbonne University, Paris, France; 3 Department of Haematology, Saint Antoine Hospital, INSERM UMR 938, Sorbonne University, Paris, France; 4 Programme de Transplantation and Therapie Cellulaire Centre de Recherche en Cancérologie de Marseille, Institut Paoli Calmettes, Marseille, France; 5 Ematologia e Terapie Cellulari, IRCCS Ospedale Policlinico San Martino, Genoa, Italy; 6 Hospital Clínico Servicio de Hematología, Salamanca, Spain; 7 Transplantation Unit Department of Oncology and Haematology, IRCCS Humanitas Research Hospital, Milan, Italy; 8 Dipartimento di Diagnostica per Immagini, Radioterapia Oncologica ed Ematologia, Fondazione Policlinico Universitario A. Gemelli IRCCS, Rome, Italy; 9 Sezione di Ematologia, Dipartimento di Scienze Radiologiche ed Ematologiche, Università Cattolica del Sacro Cuore, Rome, Italy; 10 Hematology Hospital GU Gregorio Marañon, Instituto de Investigacion Sanitaria Gregorio Marañon, Medicina UCM, Madrid, Spain; 11 Bone Marrow Transplant Unit, Medicana International Hospital Istanbul, Istanbuls, Turkey; 12 Department of Haematology, Hammersmith Hospital, Imperial College, London, UK; 13 Raisa Gorbacheva Memorial, Research Institute for Paediatric Oncology, Hematology, and Transplantation, First State Pavlov Medical University of St. Petersburg, St. Petersburg, Russia; 14 SSD Trapianto di Cellule Staminali, AOU Citta’ Della Salute e della Scienza, Turin, Italy; 15 Hospital U. Marqués de Valdecilla, Servicio de Hematología-Hemoterapia, Santander, Spain; 16 Department Hematology and Stem Cell Transplant, Dél-pesti Centrumkórház – Országos Hematológiai és Infektológiai Intézet, Budapest, Hungary; 17 Department of Hematology and Oncology, Augsburg University Hospital, Augsburg, Germany; 18 Service d’Hématologie Clinique et Thérapie Cellulaire, Hôpital Saint-Antoine, AP-HP, Sorbonne University, and INSERM UMRs 938, Paris, France; 19 Hematology Department, Hospital Universitari Politècnic La Fe, Valencia, Spain; 20 Bone Marrow Transplantation Program, Department of Internal Medicine, American University of Beirut, Beirut, Lebanon; 21 Department of Bone Marrow Transplantation and Onco-Hematology, Maria Sklodowska-Curie National Research Institute of Oncology, Gliwice Branch, Gliwice, Poland; 22 Ospedale San Raffaele, Haematology and BMT, Milan, Italy

**Correction : Journal of Hematology & Oncology (2023) 16:58** 10.1186/s13045-023-01450-4

The original article [1] incorrectly presents Affiliation 7. The correct affiliation can be viewed ahead in this Correction article:

^7^Transplantation Unit Department of Oncology and Haematology, IRCCS Humanitas Research Hospital, Milan, Italy.
