# Supplementary material for: Non-T-depleted haploidentical transplantation with post-transplant cyclophosphamide in patients with secondary versus de novo AML in first complete remission: a study from the ALWP/EBMT
Source: J Hematol Oncol. 2023 May 29;16:58. doi: 10.1186/s13045-023-01450-4 (PMC10226209; doi:10.1186/s13045-023-01450-4)
Supplement: Supplementary file 1 — Additional file 1. Contributing centers and Supplemental Tables. [file 13045_2023_1450_MOESM1_ESM.docx]

**Supplementary Appendix: Contributing Centers**

Programme de Transplantation&Therapie Cellulaire, Centre de Recherche en Cancérologie de Marseille, Institut Paoli Calmettes, Marseille, France; IRCCS Ospedale San Martino, Department of Haematology II, Genova, Italy; Hopital Saint Antoine, Department of Hematology, Paris, France;Hospital Clínico, Servicio de Hematología, Salamanca, Spain;Istituto Clinico Humanitas, Transplantation Unit, Department of Oncology and Haematology, Milano, Italy;Ospedale San Raffaele s.r.l., Haematology and BMT, Milano, Italy;Universita Cattolica S. Cuore, Istituto di Ematologia, Rome, Italy; Hospital Gregorio Marañón, Sección de Trasplante de Medula Osea, Madrid, Spain;Medicana International Hospital Istanbul, Bone Marrow Transplant Unit, Istanbul, Turkey;Imperial College, Department of Haematology, Hammersmith Hospital, London, United Kingdom;First State Pavlov Medical University of St. Petersburg, Raisa Gorbacheva Memorial Research Institute for Paediatric Oncology, Hematology, and Transplantation, St Petersburg, Russia; S.S.C.V.D Trapianto di Cellule Staminali, A.O.U Citta della Salute e della Scienza di Torino, Torino, Italy; Hospital U. Marqués de Valdecilla, Servicio de Hematología-Hemoterapia, Santander, Spain; Dél-pesti Centrumkórház –, Országos Hematológiai és Infektológiai Intézet, Dept. Haematology and Stem Cell Transplant, Budapest, Hungary;Hopital St. Louis, Dept.of Hematology - BMT, Paris, France;Turku University Hospital, TD7 (Stem Cell Transplant Unit), Turku, Finland;Institute of Hematology and Blood Transfusion, Servicio de Hematología, Prague, Czech Republic;Département d`Oncologie, Service d`Hématologie, Hôpitaux Universitaires De Genève, Geneva, Switzerland;CHU Bordeaux, Hôpital Haut-leveque, Pessac, France;CHU de Lille, LIRIC, INSERM U995, Université de Lille, Lille, France;Department of Internal Medicine, American University of Beirut Medical Center, Beirut, Lebanon;Charles University Hospital, Dept. of Hematology/Oncology, Pilsen, Czech Republic;Hospital Clinic, Institute of Hematology & Oncology, Dept. of Hematology, Barcelona, Spain;Universite Paris IV, Hopital la Pitié-Salpêtrière, Hematologie Clinique, Paris, France;ASST GRANDE OSPEDALE METROPOLITANO NIGUARDA, Hematology Department, Milano, Italy;Grande Ospedale Metropolitano Bianchi Melacrino Morelli - Centro Unico Trapianti A. Neri, Reggio Calabria, Italy;University Hospital Eppendorf, Bone Marrow Transplantation Centre, Hamburg, Germany;European Institute of Oncology, Institute of Haematology, Milano, Italy;Hospital Ramón y Cajal, Servicio de Hematología, Madrid, Spain;University Hospital La Fe, Hematology Department (Torre F, Planta 7), Valencia, Spain;Klinikum Augsburg, II Medizinische Klinik, Augsburg, Germany;Goethe-Universitaet, Medizinische Klinik II, Hämatologie, Medizinische Onkologie, Frankfurt Main, Germany;King Faisal Specialist Hospital & Research Centre, Oncology (Section of Adult Haematolgy/BMT), Riyadh, Saudi Arabia;University Hospital Maastricht, Dept. Internal Med.Hematology /Oncology, Maastricht, Netherlands;Centre Hospitalier Lyon Sud, Service Hematologie, Lyon, France;Universitaetsklinikum Dresden, Medizinische Klinik und Poliklinik I, Dresden, Germany;Fondazione IRCCS Policlinico San Matteo, Pavia, Italy;Univ. La Sapienza, Dip. Biotecnologie Cellulari ed Ematologia, Rome, Italy;CHU Nantes, Dept. D`Hematologie, Nantes, France;Arcispedale S. Maria Nuova, Unita Operativa Ematologia, Reggio Emilia, Italy;Demiroglu Bilim University Istanbul Florence Nightingale Hospital, Hematopoietic SCT Unit, Istanbul, Turkey;Medizinische Universitaet Wien, Klinik fuer Innere Medizin I, Knochenmarktransplantation, Vienna, Austria;Hopital Jean Minjoz, Service d`Hématologie, Besancon, France;ASST Papa Giovanni XXIII, Hematology and Bone Marrow Transplant Unit, Bergamo, Italy;Azienda Ospedaliero Universitaria di Udine, Division of Hematology, Udine, Italy;Azienda Ospedali Riuniti di Ancona, Department of Hematology, Ancona University, Ancona, Italy;H SS. Antonio e Biagio, Haematology Department, Alessandria, Italy; USD Trapianti di Midollo, Adulti, Universita di Brescia, Brescia, Italy;University Hospital, Hematology, Basel, Switzerland;Institut de Cancerologie Lucien Neuwirth, Service d`Hematologie Clinique, Saint Etienne, France;Hospital Santa Creu i Sant Pau, Hematology Department, Barcelona, Spain;ALBERTS CELLULAR THERAPY, Netcare Pretoria East Hospital, Pretoria, South Africa;Klinikum Grosshadern, Med. Klinik III, Munich, Germany;AORMN Hospital, Hematology & Transplant Centre, Pesaro, Italy;Hospital Sirio-Libanes, Hematology Bone Marrow Transplant Unit, Sao Paulo, Brazil;CHU Grenoble Alpes - Université Grenoble Alpes, Service d`Hématologie, Grenoble, France;Azienda Ospedaliera Universitaria Careggi, Cell Therapy and Transfusion Medicine Unit, Firenze, Italy;Evangelismos Hospital, Division of Hematology, BMT Unit, Athens, Greece;University of Amiens: CHU Amiens, Service d`Hematologie, Amiens, France;Klinikum Frankfurt (Oder) GmbH, Medizinische Klinik I, Frankfurt Oder, Germany;University Hospital, Dept. of Bone Marrow Transplantation, Essen, Germany; Hopital La Miletrie, Bone Marrow TransplantUnit, Clinical Hematology, Poitiers, France;Fondazione IRCCS - Ca’ Granda, Ospedale Maggiore Policlinico IRCCS, Milano, Italy;Baskent University Hospital, Haematology Division, BMT Unit, Haemaology Reserach Laboratory, Adana, Turkey;Anadolu Medical Center Hospital, Bone Marrow Transplantation Department, Kocaeli, Turkey;University Medical Center Groningen (UMCG), Dept. of Hematology, Groningen, Netherlands;CHRU Nancy, Vandoeuvre les nancy, Meurethe et Moselle, Vandoeuvre Nanc, France;University of Napoli, `Federico II` Medical School, Napoli, Italy;University Medical Center Mainz, Department of Hematology, Oncology and Pneumology,, Mainz, Germany;Unita Operativa di Ematologia e Trapianto di cellule staminali, Presidio Ospedaliero Vito Fazzi, Lecce, Italy;CHU Lapeyronie, Département d`Hématologie Clinique, Montpellier, France;Oslo University Hospital, Rikshospitalet, Clinic for Cancer Medicine, Hematology Dept., Section for Stem Cell Transplantation, Oslo, Norway; Hosp. Reina Sofia, Córdoba Hospital, Department of Hematology, Cordoba, Spain; University Hospital Center Rebro, Zagreb, Croatia; Fundación Jiménez Díaz, Hematología, Madrid, Spain;U.O.D Trapianti di midollo osseo, A.O.R Villa Sofia-Cervello, Palermo, Italy; HUCH Comprehensive Cancer Center, Stem Cell Transplantation Unit, Helsinki, Finland; CHU Nice - Hôpital de l`ARCHET I, Hematologie Clinique, Nice, France;George Papanicolaou General Hospital, Haematology Department / BMT Unit, Thessaloniki, Greece;Az. Ospedaliera S. Croce e Carle, Division of Hematology, Cuneo, Italy; Gustave Roussy Cancer Campus, BMT Service, Department of Hematology, Villejuif, France; Techniciens d`Etude Clinique suivi de patients greffes, Nouvel Hopital Civil, Strasbourg, France; Ospedale La Maddalena - Dpt. Oncologico, Unità Operativa di Oncoematologia e, Trapianto di Midollo, Palermo, Italy; Hospital Morales Meseguer, Unidad de Trasplante de Médula Osea, Murcia, Spain; CHU CAEN, Institut d’hématologie de Basse-Normandie, Caen, France; Hospital Clínico de Valencia, Servicio de Hematología, Valencia, Spain; Hospital Univ. 12 de Octubre, Servicio de Hematología, Madrid, Spain; Ospedale San Gerardo, Clinica Ematologica dell`Universita Milano-Biocca, Monza, Italy;Addenbrookes Hospital, Department of Haematology, Cambridge, United Kingdom;Universitair Ziekenhuis Brussel, Division of Clinical Hematology, Brussels, Belgium;A.O.R.N. `SAN.G MOSCATI`, Ematologia, Avellino, Italy;Azienda Ospedaliero Universitaria Pisana, Unità Operativa Ematologia, Pisa, Italy;University Hospital Gasthuisberg, Dept. of Hematology, Leuven, Belgium; Cliniques Universitaires St. Luc, Dept. of Haematology, Brussels, Belgium;Adult HSCT unit, Northern Centre for Bone Marrow Transplantation, Freeman Hospital, Newcastle Tyne, United Kingdom;Hannover Medical School, Department of Haematology, Hemostasis, Oncology, and Stem Cell Transplantation, Hannover, Germany;U.O.S.A Centro Trapianti e Terapia Cellulare, Azienda Ospedaliera Universitaria Senese, Policlinico S.Maria alle Scotte, Siena, Italy;King Hussein Cancer Centre, Queen Rania Street - Aljubiha, Amman, Jordan;Elisabethinen-Hospital, I. Internal Department, Linz, Austria;ICO-Hospital Universitari Germans Trias i Pujol, Cattedra e Servizio di Ematologia, Badalona, Spain;U.O. Ematologia con Trapianto, Azienda Ospedaliero Universitaria Policlinico Bari, Bari, Italy;CHRU, Service des Maladies du Sang, Angers, France;S. Bortolo Hospital, Department of Hematology, Vicenza, Italy;University Hospital Erlangen, Dept. of Internal Medicine 5, Erlangen, Germany;Hôpital Necker, Service Hematologie Adulte, Paris, France;Hôpital Henri Mondor, Sve d` Hematologie, Creteil, France;Bristol Royal Hospital for Children, Dept. of Paediatric Oncology/BMT, Bristol, United Kingdom;Hospital Regional de Málaga, Servicio de Hematología, Malaga, Spain;Philipps Universitaet Marburg, University Hospital Giessen and Marburg, Campus Marburg, Baldingerstr., Marburg, Germany;AZ Delta, Hematology Dept., Roeselare, Belgium;Centre Hospitalier Universitaire de Rennes, Service d`Hematologie Clinique Adulte, Rennes, France;University of Liege, Dept. of Hematology, Liege, Belgium;Medical University of Gdansk, University Hospital, Dept. of Haematology and Transplantology, Gdansk, Poland;Centro Trapianti Unico Di CSE Adulti e Pediatrico A. O Brotzu, Cagliari, Italy;CHRU Limoges, Service d`Hématologie Clinique, Limoges, France;University College London Hospital, Department of Haematology, London, United Kingdom;Ospedale Civile, Dipartimento Oncologico Ematologico, Pescara, Italy;Klinikum Karlsruhe gGmbH, III. Med. Klinik, Haematologie, Onkologie, Karlsruhe, Germany;Hospital San Maurizio, Dept. of Hematology - BMT Unit, Bolzano, Italy;Martin-Luther-Universitaet Halle-Wittenberg, Klinik für Innere Medizin IV, Halle, Germany;University of Heidelberg, Medizinische Klinik u. Poliklinik V, Heidelberg, Germany;Hospital Universitario Central de Asturias, C/Celestino Villamil s/n, Oviedo, Spain;Institute of Hematology and Transfusion Medicine, Warsaw, Poland;Kings College Hospital, Dept. of Haematological Medicine, King`s Denmark Hill Campus, London, United Kingdom;Ospedale San Carlo, Dip. Ematologia, Potenza, Italy; Centre Henri Becquerel, Hematology, Rouen, France; Antwerp University Hospital (UZA), Dept. of Hematology, Antwerp Edegem, Belgium;Universitaetsmedizin Mannheim, III. Medizinische Klinik, Einheit für Stammzelltransplantation, Mannheim, Germany; Karolinska University Hospital, Dept. of Hematology, Stockholm, Sweden; Institut Jules Bordet, Experimental Hematology, Brussels, Belgium; Bologna University, S.Orsola-Malpighi Hospital, Institute of Hematology & Medical, Oncology L & A Seràgnoli, Via Massarenti 9, Bologna, Italy;CHU ESTAING, Service d’hématologie clinique Adulte et pédiatrie, Clermont Ferr, France;Ospedale S. Camillo-Forlanini, Dept. of Hematology and BMT, Rome, Italy;Fundeni Clinical Institute, Bucharest, Romania;Department of Bone Marrow Transplantation and Oncohematology, Maria Sklodowska-Curie National Research Institute of Oncology, Oncology Center, Gliwice Branch, Wybrzeze Armii Kr, Gliwice, Poland;University of Cape Town Faculty of Health Sciences, Division of Clinical Haematology, Cape Town, South Africa;IRCCS, Casa Sollievo della Sofferenza, Departement of Hemato-Oncology, Stem Cell Transplant Unit, S-Giovanni Rot, Italy;Hospital de Gran Canaria `Dr Negrin`, Servicio de Hematología y Hemoterapia, Las Palmas, Spain;CHU - Institut Universitaire du Cancer Toulouse, Oncopole, I.U.C.T-O, Toulouse, France; C.H.R.U de Brest, Service Onco-Hematologie, Brest, France; Nottingham University, Hucknall Road, Nottingham, United Kingdom;Clinica Puerta de Hierro, Servicio de Hematologia y Hemoterapia, Madrid, Spain;Clínica Universitaria de Navarra, Area de Terápia Celular, Unidad de Trasplante Hemopoyético, Pamplona, Spain;Institut Catalá d`Oncologia– Hospital Duran i Reynals, L`Hospitalet de Llobregat, Barcelona, Spain;University of Freiburg, Dept. of Medicine -Hematology, Oncology, Freiburg, Germany; Gazi University Faculty of Medicine, Hematology, Ankara, Turkey; Klinik fuer Innere Medzin III, Universitätsklinikum Ulm, Ulm, GermanyNijmegen Medical Centre, Department of Hematology, Nijmegen, Netherlands;Univ. of Parma, Cattedra di Ematologia, Centro Trapianti Midollo Osseo, Parma, Italy; University Hospital Innsbruck, Internal Medicine V (Hematology & Oncology), Innsbruck, Austria; Hopital Bretonneau, Service d`Oncologie Médicale, Tours, France;Research Committee - University of Patras, University Campus - Building A, Patras, Greece;Sahlgrenska University Hospital, Center for Hematopoietic Cell Transplantation, Hematology Section, Goeteborg, Sweden;Rambam Medical Center, Dept. of Hematology & BMT, Haifa, Israel;King Fahad Specialist Hospital, Adult Hematology and HSCT department, Dammam, Saudi Arabia;King Abdul - Aziz Medical City, Riyadh, Saudi Arabia;Ospedale Dell`Angelo, Hematology Department, Venezia, Italy;A.Z. Sint-Jan, Dept. of Hematology, Brugge, Belgium;Azienda Ospedaliero Universitaria di Modena Policlinico, Ematologia, Modena, Italy;Klinikum Rechts der Isar, III Med Klinik der TU, Munich, Germany;Hospital Vall d`Hebron, Unidad de Adultos, Barcelona, Spain;Cardarelli Hospital, Division of Hematology & SCT Unit, Napoli, Italy;Policlinico G.B. Rossi, Divisione di Ematologia, Unità di TMO, Verona, Italy;University of Debrecen Clinical Center, Department of Internal Medicine, Debrecen, Hungary;Hôpital D`instruction des Armées (HIA) PERCY, Service d`Hématologie, Clamart, France;Hospital Universitario La Paz, Hematologia-Oncologia, Madrid, Spain;University Hospital, Dept. of Hematology, Linkoeping, Sweden;King Faisal Specialist Hospital and Research Center, Department of Oncology, Jeddah, Saudi Arabia;Mazzoni Hospital, Haematology Service, Ascoli Piceno, Italy;University Hospital Ostrava, Department of Haematology, Ostrava, Czech Republic;Leiden University Hospital, BMT Centre Leiden, Leiden, Netherlands;Bone Marrow Transplant Unit L 4043, National University Hospital, Rigshospitalet, Copenhagen, Denmark;Hospital de la Princesa, Department of Hematology, Madrid, Spain;Birmingham Heartlands Hospital, Department of Haematology, Birmingham, United Kingdom;Onco-Ematologia Pediatrica, Centro Trapianti Cellule Staminali, Ospedale Infantile Regina Margherita, Torino, Italy;LKH - University Hospital Graz, Division of Haematology, Graz, Austria;Hospital Universitario Virgen de la Arrixaca, Ctra. Madrid - Cartagena, Murcia, Spain; ZNA, Lange Beeldekensstraat 267, Antwerp, Belgium;Ankara Bayindir Hospital, Haematology BMT, Ankara, Turkey;National Center for Cancer Care & Research, Hematology, Doha, Qatar;University of Cologne, I. Dept. of Medicine, Cologne, Germany;Unidad de Ensayos Clínicos de Hematología Pabellón A, bajo., Complejo Hospitalario de Navarra, Pamplona, Spain;University Med. Center, Department of Hematology, Ljubljana, Slovenia;Umea University Hospital, Hematology, Umea, Sweden;Ghent University Hospital, Haematology, Gent, Belgium;Hospital Universitario Virgen del Rocío, Servicio de Hematologia y Hemoterapia, Servicio Andaluz de Salud, Sevilla, Spain;University of Saarland, University Hospital, Dept. of Internal Med., BMT Unit, Homburg, Germany;Sezione di Ematologia, Dipartimento di Medicina Clinica e Sperimentale, Università di Perugia, Perugia, Italy;Perrino Hospital, Dept. of Haematology, SS 7, Brindisi, Italy;Federal Centre of Heart, Blood and Endocrinology, Dept. of Hematology, St Petersburg, Russia;Medical School University of Salerno, AOU San Giovanni di Dio e Ruggi D´Aragona Hospital, Hematology and Hematopoietic SCT Center, Salerno, Italy;FOSCAL-UNAB, Urbanización El Bosque, Floridablanca, Floridablanca, Colombia;Instituto de Cancerologia S.A, Medellin, Medellin, Colombia;Universitaet Bonn, Medizinische Klinik III, Bonn, Germany;Robert_Bosch_Krankenhaus, Abt. Hämatologie / Onkologie, Stuttgart, Germany;Tel Aviv Sourasky Medical Center, Blood and Bone Marrow Transplantation, Tel Aviv, Israel;Hospital Guglielmo da Saliceto, Oncology and Hematology Department, Piacenza, Italy;University Hospital, Clinic of Hematology, Zurich, Switzerland;Royal Marsden Hospital, Leukaemia Myeloma Units, London, United Kingdom;Universitaet Tuebingen, Medizinische Klinik, Tuebingen, Germany;Academisch Ziekenhuis bij de Universiteit, van Amsterdam, Emma Kinderziekenhuis, Amsterdam, Netherlands;Hadassah University Hospital, Dept. of Bone Marrow Transplantation, Jerusalem, Israel;University Hospital, Dept. of Medicine, Uppsala, Sweden;Department of Haematology, University Hospital of Wales, Cardiff, United Kingdom;Ospedale Nord, Institute of Haematology, Taranto, Italy;University Hospital Aachen, Dept. of Oncology, Hematology and SCT, Medizinische Klinik IV, Aachen, Germany;Sahyadri Speciality Hospital, Department of Haematology & BMT, Pune, India;University Hospital Birmingham NHSTrust, Queen Elizabeth Medical Centre, Edgbaston, Dept. of Haematology, Birmingham, United Kingdom;Medical Clinic and Policinic 1, Hematology and Cellular Therapy, University hospital Leipzig, Leipzig, Germany;Hospital Álvaro Cunqueiro - Complejo Hospitalario Universitario de Vigo, Servicio de Hematología, Vigo, Spain;Singapore General Hospital, Singapore, Singapore;HELIOS Klinikum Berlin-Buch, Klinik für Hämatologie und Stammzelltransplantation, Berlin, Germany;Universitaetsklinikum Goettingen, Abteilung Hämatologie und Onkologie, Goettingen, Germany;Hospital Univ. Virgen de las Nieves, Servicio de Hematología, Granada, Spain;Hospital Clinico Universitario, Servicio de Hematología, S de Compostela, Spain;Ankara University Faculty of Medicine, Dept. of Hematology, Adult Stem Cell Transplantation Unit, Ankara, Turkey;Klinikum Nuernberg, 5. Medizinische Klinik, BMT-Unit, Nuernberg, Germany;Shariati Hospital, Hematology-Oncology and BMT Research, Teheran, Iran;Hospital C. Panico, Hematology, Tricase Lecce, Italy;Fundació Institut d`Investigació Sanitària Illes Balears – IdISBa, Hospital Universitari Son Espases. Edifici “S”. 1ª Planta, Palma Mallorca, Spain;Charles University Hospital, 4th Department of Internal Medicine - Hematology, Hradec Kralove, Czech Republic;Tor Vergata¨ University of Rome, Stem Cell Transplant Unit, Policlinico Universitario Tor Vergata, Rome, Italy;Ýstanbul Tip Fakultesi, Iç Hastaliklari ABD, Kemik iliði nakil unitesi, CAPA, Istanbul, Turkey;Sheffield Teaching Hospitals NHS Trust, Royal Hallamshire Hospital, Sheffield, United Kingdom;University Regensburg, Dept. of Hematology and Oncology, Regensburg, Germany;Azienda Ospedaliero Universitaria, Policlinico S.Orsola-Malpighi Bologna, Bologna, Italy;Ospedale Policlinico, Programma di Trapianto Emopoietico Misto e Metropolitano Di Catania, Catania, Italy;National Research Center for Hematology, Bone Marrow Transplantation, Moscow, Russia;First Affiliated Hospital of Soochow University, Department of Hematology, Suzhou, China;Koç University Hospital, Bone Marrow Transplantation, Istanbul, Turkey;St. Franziskus Hospital, Medizinische Klinik I, Flensburg, Germany;Meyer University Children Hospital, Pediatric Stem Cell Transplant Center, Firenze, Italy

**Supplemental Table S1**. Conditioning regimens

|  | **Overall (n=1711)** | **de novo (n=1480)** | **sAML (n=231)** |
| --- | --- | --- | --- |
| Details |  |  |  |
| TBF | 848 (49.6%) | 742 (50.2%) | 106 (45.9%) |
| BuFlu | 298 (17.4%) | 260 (17.6%) | 38 (16.5%) |
| Flu-TBI | 285 (16.7%) | 239 (16.2%) | 46 (19.9%) |
| FluTreo | 117 (6.8%) | 97 (6.6%) | 20 (8.7%) |
| FluMel | 67 (3.9%) | 57 (3.9%) | 10 (4.3%) |
| FTM | 24 (1.4%) | 21 (1.4%) | 3 (1.3%) |
| Bu-TBI | 23 (1.3%) | 20 (1.4%) | 3 (1.3%) |
| BuCy | 11 (0.6%) | 11 (0.7%) | 0 (0%) |
| Cy-TBI | 7 (0.4%) | 7 (0.5%) | 0 (0%) |
| FLAMSA-TBI | 7 (0.4%) | 4 (0.3%) | 3 (1.3%) |
| Other CT | 6 (0.4%) | 6 (0.4%) | 0 (0%) |
| FLAMSA-Treo | 4 (0.2%) | 4 (0.3%) | 0 (0%) |
| Flucy | 3 (0.2%) | 3 (0.2%) | 0 (0%) |
| FLAMSA-Mel | 3 (0.2%) | 3 (0.2%) | 0 (0%) |
| TBI-VP16 | 3 (0.2%) | 1 (0.1%) | 2 (0.9%) |
| Thiotepa_based | 2 (0.1%) | 2 (0.1%) | 0 (0%) |
| FLAMSA-Bu | 1 (0.1%) | 1 (0.1%) | 0 (0%) |
| TBI-other | 1 (0.1%) | 1 (0.1%) | 0 (0%) |
| Missing | 1 | 1 | 0 |

Abbreviations: sAML-secondary acute myeloid leukemia; TBI-total body irradiation; Mel-melphalan; Bu-busulfan; Flu-fludarabine; Cy-cytoxan; TBF-thiotepa, Bu and Flu; FTM-Flu, thiotepa, mel; CT-chemotherapy; Treo-treosulfan; FLAMSA-Flu, Amsacrine, cytarabine; VP16-etoposide

**Supplemental Table S2.** Anti-GVHD prophylaxis regimens

|  | **Overall (n=1711)** | **de novo (n=1480)** | **sAML (n=231)** |
| --- | --- | --- | --- |
| CSA+MMF | 921 (53.8%) | 797 (53.9%) | 124 (53.7%) |
| MMF+Tacro | 508 (29.7%) | 444 (30%) | 64 (27.7%) |
| MMF+Siro | 71 (4.1%) | 60 (4.1%) | 11 (4.8%) |
| Tacro | 41 (2.4%) | 36 (2.4%) | 5 (2.2%) |
| MMF | 37 (2.2%) | 32 (2.2%) | 5 (2.2%) |
| CSA | 32 (1.9%) | 30 (2%) | 2 (0.9%) |
| CSA+MTX | 23 (1.3%) | 18 (1.2%) | 5 (2.2%) |
| CSA+MMF+Tacro | 21 (1.2%) | 16 (1.1%) | 5 (2.2%) |
| CSA+MTX+MMF | 11 (0.6%) | 11 (0.7%) | 0 (0%) |
| CSA+Tacro | 7 (0.4%) | 7 (0.5%) | 0 (0%) |
| MTX+Tacro | 4 (0.2%) | 3 (0.2%) | 1 (0.4%) |
| Tacro+Siro | 2 (0.1%) | 1 (0.1%) | 1 (0.4%) |
| MTX | 1 (0.1%) | 1 (0.1%) | 0 (0%) |
| Other | 32 (1.9%) | 24 (1.6%) | 8 (3.5%) |

Abbreviations: sAML-secondary acute myeloid leukemia; CSA- cyclosporine A; MTX- methotrexate; MMF- mycophenolate mofetil; Siro- sirolimus; Tacro- tacrolimus

**Supplemental Table S3.** Matched-pair analysis: Patient, disease, and transplant matching criteria

|  | **de novo (n=410)** | **sAML (n=211)** | **P** |
| --- | --- | --- | --- |
|  |  | MDS/MPN/BMFS (n=141) |  |
|  |  | OMHD/ST (n=70) |  |
| Median follow-up (months) [quartiles] | 26.29 [23.5-29.98] | 24.68 [20.31-31.19] | 0.05 |
| Patient age (years), median (min-max) [IQR] | 60.3 (18.3-75.4) [51.6-66.4] | 60.5 (20.8-75.7) [51-66.8] | 0.65 |
| Year transplant, median (min-max) | 2019 (2011-2021) | 2019 (2010-2021) | 0.29 |
| cytogenetics |  |  |  |
| Interm | 282 (68.8%) | 145 (68.7%) | 0.99 |
| Adverse | 128 (31.2%) | 66 (31.3%) |  |
| Time diagnosis to HSCT (mo), median (min-max) [IQR] | 4.8 (1-22.3) [3.9-6.1] | 4.9 (1.3-20.5) [3.5-6.5] | 0.73 |
| HTCI |  |  |  |
| HT-CI = 0 | 188 (53%) | 73 (42.9%) | 0.002 |
| HT-CI = 1 or 2 | 78 (22%) | 29 (17.1%) |  |
| HT-CI >=3 | 89 (25.1%) | 68 (40%) |  |
| Missing | 55 | 41 |  |
| Karnofsky score |  |  |  |
| <90 | 116 (28.3%) | 63 (29.9%) | 0.68 |
| >=90 | 294 (71.7%) | 148 (70.1%) |  |
| Patient sex |  |  |  |
| Male | 229 (55.9%) | 127 (60.2%) | 0.3 |
| Female | 181 (44.1%) | 84 (39.8%) |  |
| Donor sex |  |  |  |
| male | 268 (65.4%) | 134 (63.8%) | 0.7 |
| female | 142 (34.6%) | 76 (36.2%) |  |
| Missing | 0 | 1 |  |
| Female to male combination |  |  |  |
| No F->M | 334 (81.5%) | 169 (80.1%) | 0.68 |
| F->M | 76 (18.5%) | 42 (19.9%) |  |
| Patient CMV |  |  |  |
| neg. | 81 (19.9%) | 48 (23.2%) | 0.34 |
| pos | 326 (80.1%) | 159 (76.8%) |  |
| Missing | 3 | 4 |  |
| Donor CMV |  |  |  |
| neg. | 166 (41.1%) | 88 (42.9%) | 0.66 |
| pos | 238 (58.9%) | 117 (57.1%) |  |
| Missing | 6 | 6 |  |
| Conditioning |  |  |  |
| MAC | 146 (35.6%) | 75 (35.5%) | 0.99 |
| RIC | 264 (64.4%) | 136 (64.5%) |  |
| Cell source |  |  |  |
| BM | 92 (22.4%) | 47 (22.3%) | 0.96 |
| PB | 318 (77.6%) | 164 (77.7%) |  |
| MRD pre HSCT |  |  |  |
| neg | 134 (65.4%) | 36 (61%) | 0.54 |
| pos | 71 (34.6%) | 23 (39%) |  |
| Missing | 205 | 152 |  |

Abbreviations: sAML-secondary acute myeloid leukemia; min-minimum; max-maximum; IQR-interquartile range; Interm- intermediate; MRD-measurable residual disease; F-female; M-male; CMV- cytomegalovirus; neg-negative; pos-positive; HCT-CI- hematopoietic cell transplantation-specific comorbidity index; BM-bone marrow; PB-peripheral blood; Mac-myeloablative conditioning; RIC-reduced intensity conditioning; MDS/MPN/BMF - sAML post myelodysplastic syndrome, myeloproliferative neoplasm, bone marrow failure syndrome; OMHD/ST -sAML post other malignant hematologic disorders and solid tumors

**Supplemental Table S4.** Matched-pair analysis- Transplantation Outcomes: Engraftment and acute graft versus host disease

|  | **de novo (n=410)** | **sAML (n=211)** | **P** |
| --- | --- | --- | --- |
|  |  | MDS/MPN/BMFS (n=141) |  |
|  |  | OMHD/ST (n=70) |  |
| Engraftment HSCT |  |  |  |
| Graft failure | 26 (6.6%) | 10 (5.1%) | 0.47 |
| Engrafted | 368 (93.4%) | 186 (94.9%) |  |
| Missing | 16 | 15 |  |
| Cumulative incidence of PMN>500, day 30 | 88.8%[85.2-91.5] | 87.2%[81.7-91.2] | 0.63 |
| Acute GVHD |  |  |  |
| Grade I | 71 (18%) | 36 (18.4%) | Not done |
| Grade II | 73 (18.5%) | 41 (20.9%) |  |
| Grade III | 33 (8.4%) | 9 (4.6%) |  |
| Grade IV | 4 (1%) | 4 (2%) |  |
| Present, grade unknown | 6 (1.5%) | 1 (0.5%) |  |
| No aGvHD present (Grade 0) | 208 (52.7%) | 105 (53.6%) |  |
| Missing | 15 | 15 |  |

Abbreviations: sAML-secondary acute myeloid leukemia; MDS/MPN/BMF - sAML post myelodysplastic syndrome, myeloproliferative neoplasm, bone marrow failure syndrome; OMHD/ST -sAML post other malignant hematologic disorders and solid tumors; HSCT-hematopoietic stem cell transplantation; GVHD- graft-versus-host disease; a-acute; PMN-polymorphonuclear cells

**Supplemental Table S5.** Matched-pair analysis: Conditioning regimens

|  | **de novo (n=410)** | **sAML (n=211)** |
| --- | --- | --- |
|  |  | MDS/MPN/BMFS (n=141) |
|  |  | OMHD/ST (n=70) |
| Details |  |  |
| TBF | 209 (51%) | 97 (46%) |
| Flu-TBI | 75 (18.3%) | 42 (19.9%) |
| BuFlu | 58 (14.1%) | 35 (16.6%) |
| FluTreo | 32 (7.8%) | 18 (8.5%) |
| FluMel | 19 (4.6%) | 10 (4.7%) |
| Bu-TBI | 8 (2%) | 2 (0.9%) |
| FTM | 5 (1.2%) | 2 (0.9%) |
| FLAMSA-Mel | 2 (0.5%) | 0 (0%) |
| FLAMSA-Treo | 1 (0.2%) | 0 (0%) |
| Cy-TBI | 1 (0.2%) | 0 (0%) |
| FLAMSA-TBI | 0 (0%) | 3 (1.4%) |
| TBI-VP16 | 0 (0%) | 2 (0.9%) |

1. Abbreviations: sAML-secondary acute myeloid leukemia; MDS/MPN/BMF - sAML post myelodysplastic syndrome, myeloproliferative neoplasm, bone marrow failure syndrome; OMDS/ST -sAML post other malignant hematologic disorders and solid tumors; TBI-total body irradiation; Mel-melphalan; Bu-busulfan; Flu-fludarabine; Cy-cytoxan; TBF-thiotepa, Bu and Flu; FTM-Flu, thio, mel; CT-chemotherapy; Treo-treosulfan

**Supplemental Table S6. Matched-pair analysis:** Anti-GVHD prophylaxis regimens

|  | **de novo (n=410)** | **sAML (n=211)** |
| --- | --- | --- |
|  |  | MDS/MPN/BMFS (n=141) |
|  |  | OMHD/ST (n=70) |
| CSA+MMF | 215 (52.4%) | 111 (52.6%) |
| MMF+Tacro | 129 (31.5%) | 60 (28.4%) |
| MMF+Siro | 19 (4.6%) | 10 (4.7%) |
| MMF | 11 (2.7%) | 5 (2.4%) |
| Tacro | 9 (2.2%) | 4 (1.9%) |
| CSA+MTX | 6 (1.5%) | 5 (2.4%) |
| CSA | 5 (1.2%) | 2 (0.9%) |
| CSA+MMF+Tacro | 5 (1.2%) | 4 (1.9%) |
| CSA+MTX+MMF | 2 (0.5%) | 0 (0%) |
| MTX | 1 (0.2%) | 0 (0%) |
| CSA+Tacro | 1 (0.2%) | 0 (0%) |
| MTX+Tacro | 0 (0%) | 1 (0.5%) |
| Tacro+Siro | 0 (0%) | 1 (0.5%) |
| Other | 7 (1.7%) | 8 (3.8%) |

Abbreviations: sAML-secondary acute myeloid leukemia; MDS/MPN/BMF - sAML post myelodysplastic syndrome, myeloproliferative neoplasm, bone marrow failure syndrome; OMHD/ST -sAML post other malignant hematologic disorders and solid tumors; CSA- cyclosporine A; MTX- methotrexate; MMF- mycophenolate mofetil; Siro- sirolimus; Tacro- tacrolimus

**Supplemental Table S7.** Matched per analysis: Causes of death

|  | **de novo (n=148)** | **sAML (n=69)** |
| --- | --- | --- |
| Original disease | 53 (37.3%) | 27 (40.3%) |
| Infection | 45 (31.7%) | 18 (26.9%) |
| GVHD | 12 (8.5%) | 7 (10.4%) |
| Non HSCT related | 12 (8.5%) | 7 (10.4%) |
| VOD | 5 (3.5%) | 0 (0%) |
| Cardiac toxicity | 4 (2.8%) | 1 (1.5%) |
| MOF | 3 (2.1%) | 3 (4.5%) |
| Other transp related | 3 (2.1%) | 0 (0%) |
| Other second malignancy | 2 (1.4%) | 1 (1.5%) |
| Haemorhage | 1 (0.7%) | 1 (1.5%) |
| Failure/Rejection | 1 (0.7%) | 1 (1.5%) |
| CNS toxicity | 1 (0.7%) | 1 (1.5%) |
| Missing | 6 | 2 |

Abbreviations: sAML-secondary acute myeloid leukemia; GVHD- graft-versus-host disease; HSCT-hematopoietic stem cell transplantation VOD- veno occlusive disease of the liver; MOF-multi organ failure; CNS-central nervous system
